# Methylation profiling of Epstein-Barr virus immediate-early gene promoters, *BZLF1 *and *BRLF1 *in tumors of epithelial, NK- and B-cell origins

**DOI:** 10.1186/1471-2407-12-125

**Published:** 2012-03-29

**Authors:** Lili Li, Xianwei Su, Gigi Ching Gee Choi, Ya Cao, Richard F Ambinder, Qian Tao

**Affiliations:** 1 Cancer Epigenetics Laboratory, Department of Clinical Oncology, State Key Laboratory of Oncology in South China, Sir YK Pao Center for Cancer and Li Ka Shing Institute of Health Sciences, The Chinese University of Hong Kong and CUHK Shenzhen Research Institute, Hong Kong, China; 2 Cancer Research Institute, Xiangya School of Medicine, Central South University, Changsha, China; 3 Johns Hopkins Singapore and Sidney Kimmel Comprehensive Cancer Center, Johns Hopkins School of Medicine, Baltimore, MD, USA

## Abstract

**Background:**

Epstein-Barr virus (EBV) establishes its latency in EBV-associated malignancies, accompanied by occasionally reactivated lytic cycle. Promoter CpG methylation of EBV genome plays an essential role in maintaining viral latency. Two immediate-early (IE) genes, BZLF1 and BRLF1, induce the switch from latent to lytic infection. Studies of methylation-dependent binding of BZLF1 and BRLF1 to EBV promoters have been well reported, but little is known about the methylation status of *BZLF1 *and *BRLF1 *promoters (Zp and Rp) in tumor samples.

**Methods:**

We evaluated the methylation profiles of Zp and Rp by methylation-specific PCR (MSP) and bisulfite genomic sequencing (BGS), as well as *BZLF1 *and *BRLF1 *expression by semiquantitative reverse transcription (RT)-PCR in tumors of epithelial, NK- and B-cell origins.

**Results:**

We found that both Zp and Rp were hypermethylated in all studied EBV-positive cell lines and tumors of lymphoid (B- or NK cell) or epithelial origin, while unmethylated Zp and Rp alleles were detected in cell lines expressing *BZLF1 *and *BRLF1*. Following azacytidine treatment or combined with trichostatin A (TSA), the expression of *BZLF1 *and *BRLF1 *was restored along with concomitant promoter demethylation, which subsequently induced the reactivation of early lytic gene *BHRF1 *and late lytic gene *BLLF1*.

**Conclusions:**

Hypermethylation of Zp and Rp mediates the frequent silencing of *BZLF1 *and *BRLF1 *in EBV-associated tumors, which could be reactivated by demethylation agent and ultimately initiated the EBV lytic cascade.

## Background

Epstein Barr virus (EBV) is a tumor virus associated with multiple human malignancies of lymphoid or epithelial origin, including Burkitt lymphoma (BL), Hodgkin disease (HD), nasopharyngeal carcinoma (NPC), gastric carcinoma (GsCa), nasal NK-lymphoma and posttransplant lymphoproliferative disease (PTLD), with more than 90% of adults infected in the world [1,2]. EBV has two types of infection in cells: latent or lytic. It persists in the human host as lifelong latent infection, which requires periodically reactivation of lytic genes and viral replication for maintaining its latency [3]. Two immediate-early (IE) proteins, BZLF1 (Zta) and BRLF1 (Rta), are essential to the switch from latent to lytic infection [4].

Epigenetic regulation of EBV genome is a fundamental regulatory mechanism determining different types of EBV infections in its associated tumors [5-8]. Several latent or lytic genes, including EBV nuclear antigens (EBNA-2, 3A, 3B, 3 C), latent membrane protein 1 (LMP1), IE antigens (Zta, Rta) and lytic cycle viral kinases, have been identified tightly controlled by the CpG methylation of various EBV promoters [1,5,9,10], such as W promoter (Wp), C promoter (Cp), Q promoter (Qp), F promoter (Fp), LMP1 promoters (ED-L1 and ED-L1E promoters), Z promoter (Zp) and R promoter (Rp). The precise epigenetic regulation ensures the production of viral progeny without releasing viral antigens detectable by host immune system. Meanwhile, reactivation of viral genes from latency by demethylation agents could serve as a therapeutic strategy for EBV-associated tumors [1,10-12].

Our previous work characterized the CpG methylation of EBV major latent promoters Qp, Fp and Cp by genomic sequencing [13,14]. Recent studies of IE genes have been focused on the methylation-dependent binding and activation of Zta to Rp and other viral promoters [15-17], while the overall methylation status of Zp and Rp in tumor cells still remains unclear. Here, we studied the methylation profiles of Zp and Rp in a series of EBV-positive cell lines and primary tumors of epithelial, NK- or B-cell origin. We also evaluated the effect of demethylation agent on the reactivation of *BZLF1 *and *BRLF1 *in EBV-positive cell lines.

## Methods

### Cell lines and tumor samples

B95-8 is an EBV-immortalized lymphoblastoid cell line. Rael, Akata, Wanyonyi (Wan), Raji, Namalwa, and AG876 are EBV-positive BL cell lines [13,14]. SNK6 and NK-YS are EBV-positive NK-cell lymphoma cell lines [13,14]. C666-1 is EBV-positive NPC cell line [18]. SNU719 and YCCEL1 (Rha, Tao, et al, unpublished) are EBV-positive gastric carcinoma cell lines [19,20]. All cell lines were cultivated at 37°C in RPMI 1640 supplemented with 10% fetal bovine serum (FBS), 1 mmol/L glutamine, 100 U/ml penicillin and streptomycin (Invitrogen, CA). Cell lines were maintained at 37°C in cRPMI 1640. 5-aza-dC (Sigma-Aldrich, St Louis, MO) was used at different concentrations and time to treat Rael, NK-YS and C666-1 cell lines. Other cell lines were treated with 10 μmol/L 5-aza-dC (Aza, Sigma-Aldrich, St. Louis, MO) for 3 days or further treated with 100 nmol/L trichostatin A (TSA, Cayman Chemical Co., Ann Arbor, MI) for additional ~16 h as described previously [21,22]. Archival tumor DNA samples have been previously described [21,23-25]. The study was approved by Johns Hopkins Medicine Institutional Review Board.

### Semiquantitative reverse transcription (RT)-PCR

Reverse transcription using random hexamer and RT-PCR using Go-Taq (Promega, Madison, WI) were performed as previously, with *GAPDH *as a control [26]. Sequences of primers are listed in Table 1.

**Table 1:** Sequences of primers used in this study

v*p*	*Primers*	*Sequence (5'-3')*	*Product size*	*Annealing Temp.(°C)*	*Cycles*
*RT-PCR*	*BZLF1*F	GGGAGAAGCACCTCAACCTG	255-bp	55	35
	*BZLF1*R	TTGCTTAAACTTGGCCCGGC			
	*BRLF1*F	CAAACAGACGCAGATGAGGC	443-bp	55	35
	*BRLF1*R	GCGGTGCCTATGGTGGCAGG			
	*BHRF1*H2	GTCAAGGTTTCGTCTGTGTG	213-bp	55	35
	*BHRF1*H3	TTCTCTTGCTGCTAGCTCCA			
	*BLLF1*F	GTGGATGTGGAACTGTTTCCAG	230-bp	55	35
	*BLLF1*R	CTGTATCCACCGCGGATGTCAC			
	*BART*A3	AGAGACCAGGCTGCTAAACA	232-bp	55	35
	*BART*A4	AACCAGCTTTCCTTTCCGAG			
	*GAPDH*F	GATGACCTTGCCCACAGCCT	303-bp	55	23
	*GAPDH*R	ATCTCTGCCCCCTCTGCTGA			
*MSP*	Zpm1	TCGGATTTTCGTGGGTTAATC	101-bp	64	42
	Zpm2	CGAAACTAACGTTAAAAATCCG			
	Zpu1	ATTTGGATTTTTGTGGGTTAATT	101-bp	62	40
	Zpu2	CCAAAACTAACATTAAAAATCCA			
	Rpm1	TAGGTATTTTGGATATTCGTAC	172-bp	64	42
	Rpm3	GATTTAATCCCAACCGCCG			
	Rpu1	TGTAGGTATTTTGGATATTTGTAT	78-bp	62	40
	Rpu2	CAAATCATTACTCCAAACTATCA			
*BGS*	ZpMZP1	AAAAAACACCTAATATAAATCAAA	814-bp	62	42
	ZpSC22	GTTAAAGAGGATTAGGTTTTTTT			
	RpMRP1	TCTTTTATAAACCATTAACATAAA	599-bp	60	42
	RpMRP2	GAGATTGATAGGATTATATTGT			

### DNA bisulfite treatment and methylation analysis

Bisulfite modification of DNA, methylation-specific PCR (MSP), and bisulfite genomic sequencing (BGS) were carried out as described [14,26]. Bisulfite-treated DNA was PCR amplified with strand-specific primers (for bisulfite-converted top strand of Zp and Rp) for BGS. MSP and BGS primers of Zp and Rp are listed in Table 1.

## Results

### Analysis of CpG sites in Z and R promoters

**Figure 1:**
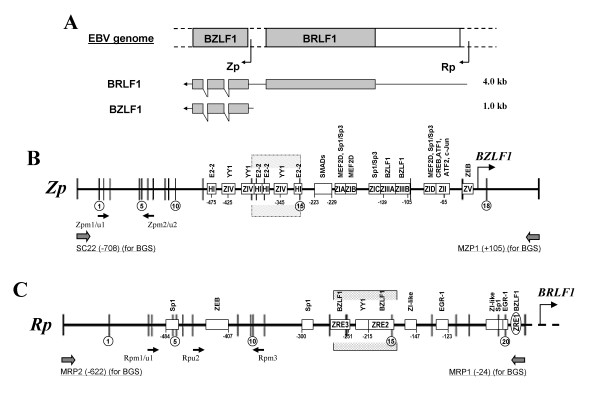
**Transcription of EBV immediate-early (IE) genes**. (A) The locations of two immediate-early (IE) genes, BZLF 1 and BRLF1, the two IE promoters, Zp and Rp, are shown. (B, C) Schematic drawing of the structure of Zp and Rp including the distribution of CpG sites, transcriptional elements and the location of analyzed MSP sites and BGS region. HI: the multiply repeated sequence motif. ZI, ZII and ZIII: three positive regulatory *cis*-acting elements. ZI motifs (ZIA, ZIB, ZIC, and ZID): AT-rich sequences. ZRE: Zta-response element. Shadowed region in Zp indicates a region not methylated in any sample. Shadowed region in Rp indicates the binding site for Zta.

The two IE transcripts derived from EBV genome are shown in Figure 1A, *BZLF1 *and *BRLF1 *are located adjacent to each other on the EBV genome. *BZLF1 *mRNA (~1.0 kb) can be transcribed from both Zp and Rp, but its protein product Zta mainly derived from Zp. *BRLF1 *mRNA (~4.0 kb) is transcribed from Rp and encodes Rta. We evaluated the distribution of CpG sites and transcriptional regulatory motifs in Zp and Rp. In Zp, only few CpG sites exist in the "ZI" and "ZII" motifs, which are the critical elements for activation of Zp by binding of several transcript factors including CREB, ATF1/2, MEF2D and Smad3/Smad4. The other negative regulatory motifs in Zp, like the proximal ZV element for binding of zinc-finger E-box binding factor (ZEB), and distal elements for binding of transcription factors YY1 and E-box binding protein (E2-2), show scattered distribution of CpG sites (Figure 1B). In Rp, two Zta-responsive elements (RpZRE2 and RpZRE3) contain several CpG sites, mainly responsible for *BRLF1 *transcription, while other regulatory elements, such as Sp1, EGR-1, contain only 1-2 CpG sites (Figure 1C). Thus, promoter CpG methylation of Zp and Rp may regulate *BZLF1 *and *BRLF1 *activation in EBV life cycle.

### Methylation status of Zp and Rp in EBV-positive cell lines of epithelial, NK- or B-cell origins

**Figure 2:**
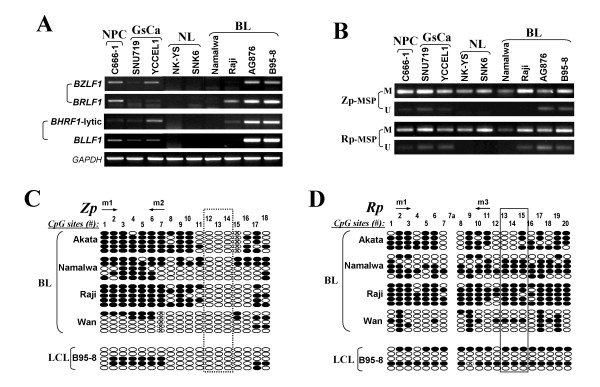
**(A, B) The expression of *BZLF1*, *BRLF1*, *BHRF1 *(lytic) and *BLLF1 *examined by RT-PCR, and methylation of Zp and Rp analyzed by MSP in EBV-positive cell lines**. M, methylated; U, unmethylated. (C, D) BGS analyses of Zp and Rp methylation status in Burkitt lymphoma (BL) and lymphoid (LCL) cell lines. Circles, CpG sites analyzed; row of circles, an individual promoter allele sequenced; Filled circle, methylated; empty circle, unmethylated. "x" circle, CpG site abolished by sequence variation or undetermined methylation status due to unconverted C in nearby CpN site.

We then examined the expression of *BZLF1 *and *BRLF1 *in EBV-positive tumor cell lines. Results showed that *BZLF1 *and *BRLF1 *were readily expressed in EBV-positive cell lines (C666-1, YCCEL1, B95-8, and AG876), with expression of early lytic gene *BHRF1 *and late lytic gene *BLLF1 *also detected in these cell lines. In addition, weak expression of *BRLF1 *was detected in Raji cells but without *BZLF1 *(Figure 2A). SUN719 showed only trace expression of *BZLF1*, *BRLF1 *and lytic *BHRF1 *but weak expression of *BLLF1*.

Next, we used methylation-specific PCR (MSP) to evaluate the promoter methylation of Zp and Rp. Primers were designed to specifically amplify a region containing the dense CpG sites in Zp and Rp. Zp and Rp methylation was detected in all EBV-positive cell lines, while unmethylated alleles were mainly seen in cell lines expressing *BZLF1 *and/or *BRLF1 *(Figure 2B). To further confirm the MSP results and characterize the methylation status of Zp and Rp in more detail, we performed high-resolution bisulfite genome sequencing (BGS) analysis of 18 CpG sites and 20 CpG sites spanning Zp and Rp, respectively. In EBV-positive BL cell lines Real, Akata, Namalwa and Raji, dense methylation was observed at both Zp and Rp, while relatively more unmethylated alleles in Zp and Rp were detected in B95-8 and Wan cell lines which are with high level of spontaneous EBV lytic replication [27,28]. Notably, three CpG sites (#12-14) in Zp were always unmethylated (Figure 2C, D, Table 2). These results suggest that promoter methylation is closely related to the transcriptional repression of *BZLF1 *and *BRLF1 *in EBV-positive cell lines.

**Table 2:** Methylation status of Zp and Rp in EBV-associated cell lines and tumors

EBV-associated cell lines and tumors	Zp methylation status (Methylated CpG sites, %)	Rp methylation status (methylated CpG sites, %)
**Cell lines**		
Akata	67%	59%
B95-8	17%	36%
C666-1	75%	99%
Namalwa	46%	53%
Raji	60%	87%
Rael	67%	83%
Wan	18%	29%
**Nude mice-passaged tumors**		
C15	68%	96%
C18	56%	59%
**Tumors**		
NPC		
NPC1	58%	97%
NPC2	92%	88%
NPC12	70%	87%
BL		
BL3	62%	80%
BL5	53%	82%
BL10	67%	82%
PTLD		
PTLD2	32%	64%
PTLD4a	44%	85%
PTLD8	22%	70%
PTLD10	47%	73%
PTLD19	53%	80%
PTLD20	75%	80%

### Zp and Rp methylation in EBV-positive tumors

EBV-positive tumors of epithelial or lymphoid origin including NPC, BL and PTLD samples, as well as two nude mice-passaged undifferentiated

**Figure 3:**
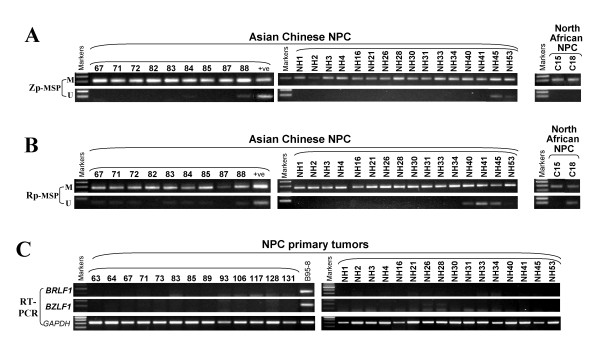
**(A, B, C) Representative MSP analysis of Zp and Rp methylation**. M, methylated; U, unmethylated. NPC, nasopharyngeal carcinoma. (D) RT-PCR analysis of *BZLF1 *and *BRLF1 *expression in primary NPC.

NPC tumors (C15 and C18) were studied. MSP analysis showed that Zp and Rp methylation was detected in virtually all 38 NPC tumors, with unmethylated Zp and Rp only detected in rare cases (Figure 3A, B), well correlated with the general silencing of *BZLF1 *and *BRLF1 *in NPC (Figure 3C).

**Figure 4:**
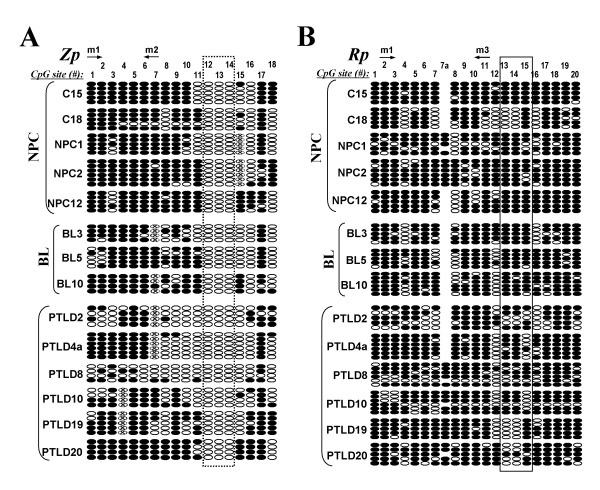
**(A, B) Methylation profiling of Zp and Rp of multiple EBV-associated tumors**. NPC, nasopharyngeal carcinoma; BL, Burkitt lymphoma; PTLD, posttransplant lymphoproliferative disease. Circles, CpG sites analyzed; row of circles, an individual promoter allele sequenced; Filled circle, methylated; empty circle, unmethylated. "x" circle, CpG site abolished by sequence variation or undetermined methylation status due to unconverted C in nearby CpN site.

We further studied the detailed methylation profiles of Zp and Rp by BGS in EBV-positive tumors. Results revealed that both Zp and Rp were heavily methylated in all studied samples, but relatively more unmethylated alleles in Zp were observed in PTLD patients (Figure 4A, B). Again, we observed that the three CpG sites (#12-14) in Zp were unmethylated in virtually all studied tumors (Figure 4A). These results are summarized in Table 2.

### Restoration of *BZLF1 *and *BRLF1 *expression by demethylation in EBV-positive cell lines

**Figure 5:**
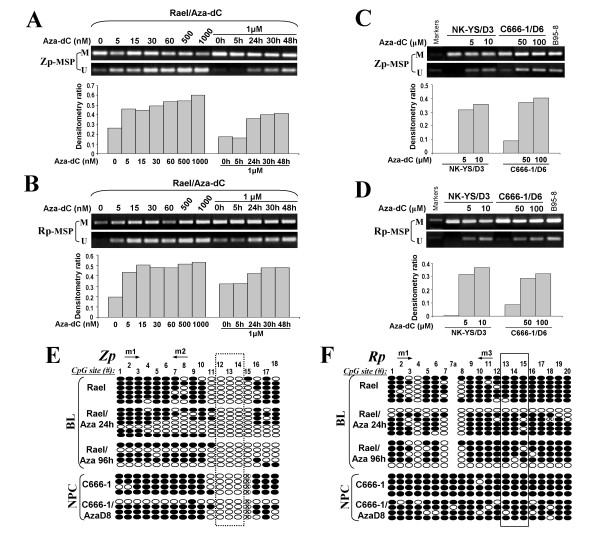
**(A-D) Pharmacologic demethylation with 5-aza-dC at indicated concentrations and time points analyzed by MSP in Rael, NK-YS and C666-1 cell lines**. Each bottom panel is the plotted densitometry ratios as % of methylation (U/U + M) of Zp and Rp. M, methylated; U, unmethylated. (E, F). Detailed BGS analysis of demethylation after 5-aza-dC treatment in Rael and C666-1 cell lines. "x" circle, CpG site abolished by sequence variation or undetermined methylation status due to an unconverted C in nearby CpN site.

**Figure 6:**
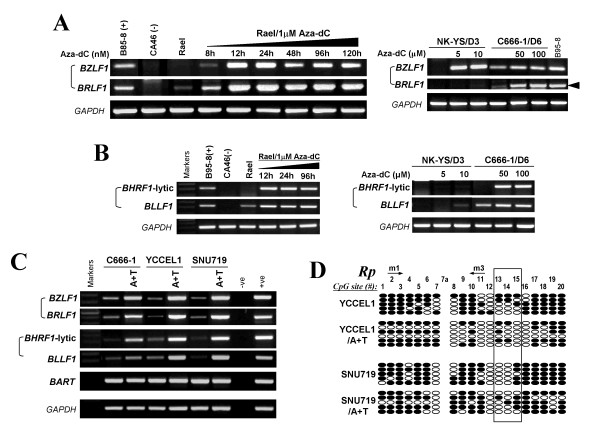
**(A, B, C) Restoration of *BZLF1 *and *BRLF1 *expression, as well as *BHRF1 *(lytic) and *BLLF1 *by 5-aza-dC treatment or combined with HDACi-TSA analyzed by RT-PCR**. *BART *as an internal control for EBV latent gene expression. (D) Methylation profiling of Rp by detailed BGS analysis of demethylation after 5-aza-dC treatment with TSA in YCCEL1 and SNU719 cell lines.

To determine whether methylation directly mediates the transcriptional repression of *BZLF1 *and *BRLF1*, Rael, NK-YS and C666-1 were treated with 5-aza-2'-deoxycytidine (5-aza-dC), a DNA methyltransferase inhibitor. Rael treated with 5-aza-dC at different concentrations (0.005, 0.015, 0.03, 0.06, 0.5 and 1 μM) for 72 h. MSP analysis showed that unmethylated Rp and Zp alleles were increased in a dose-dependent manner, with significant demethylation of Rp and Zp observed by treatment with 1 μM 5-aza-dC. Subsequently, 1 μM of 5-aza-dC was chosen to evaluate the demethylation of Zp and Rp for different indicated times (5 h, 24 h, 30 h, 48 h) in Rael cell line. It was found that both Zp and Rp were demethylated in a time-dependent manner (Figure 5A, B). Similarly, NK-YS and C666-1 cells treated with 5-aza-dC at different concentrations showed obvious demethylation of Zp and Rp, compared with untreated cells (Figure 5C, D). Concomitantly, Zp and Rp alleles were partially demethylated after 5-aza-dC treatment by high-resolution BGS analysis (Figure 5E, F, Table 2),

We further found that after 5-aza-dC demethylation or combined with the histone deacetylase inhibitor (HDACi) trichostatin A (TSA) treatment, the expression of *BZLF1 *and *BRLF1 *was dramatically increased in EBV-positive epithelial cell lines, as measured by RT-PCR, along with obviously induced expression of early lytic gene *BHRF1 *and late lytic gene *BLLF1 *(Figure 6A, B, C), suggesting the initiation of EBV-lytic cascade by DNA demethylation. We further performed the profiling of Rp methylation after 5-aza-dC treatment with TSA in YCCEL1 and SNU719. Detailed mapping of Rp by BGS analysis revealed Rp demethylation after 5-aza-dC treatment combined with TSA as expected, while the critical sites of Rp (CpG site #13-15) still maintain partially methylated required for Zta activation (Figure 6D), indicating epigenetic-mediated silencing of *BZLF1 *and *BRLF1 *in EBV-associated epithelial tumors.

## Discussion

This study characterized the CpG methylation profiles of EBV immediate-early lytic promoters Zp and Rp in cell lines and tumors of epithelial or lymphoid origin, and further evaluated the reactivation of *BZLF1 *and *BRLF1 *by demethylation treatment. We found that Zp and Rp were frequently methylated in all EBV-positive cell lines and tumors, whereas unmethylated Zp and Rp were mainly present in EBV-positive cell lines with lytic activities, along with the expression of *BZLF1 *and *BRFL1*. We did not observe major difference in Zp and Rp methylation in cell lines/or tumors of epithelial, NK- or B-cell origin.

We also demonstrated that demethylation of Zp and Rp by treatment with 5-aza-dC alone or combined with TSA resulted in the re-expression of *BZLF1 *and *BRLF1 *and activation of EBV lytic cycle. It has been identified that DNA synthesis inhibitors have no effect on DNA methylation by using four different inhibitors of DNA replication [29]. Although DNA synthesis inhibitors will delay some of the cytosine methylation, all delayed DNA methylation will be finally completed prior to the subsequent S phase. Thus, in our study, DNA methylation inhibitors are mainly responsible for Zp and Rp demethylation and initiation of lytic cascade, while other events indirectly leading to EBV reactivation also cannot be ruled out.

In EBV-positive cell lines except for B95-8, Wan and AG876, only very few % of cells or no cell undergoing spontaneous lytic infection without much lytic virion DNA, thus the methylation status of Zp and Rp detected represents the latent viral genome but not the virion genome, while in B95-8, Wan and AG876 cell lines with significant % of cells undergoing spontaneous lytic infection, the methylation status detected probably represents both latent and viral genomes (Figure 2C, D). Similarly, in EBV-positive tumors with rare cell undergoing spontaneous lytic infection, methylation status represents the latent genome [1]. In our study, lower level of methylation was only observed in B95-8 and Wan cell lines, but not in other EBV-positive cell lines and primary tumors, consistent with the high level of spontaneous EBV lytic replication only in B95-8 and Wan.

The existence of a small proportion of cells expressing viral lytic genes is essential for the success maintenance of EBV latency in host cells with a highly methylated viral genome [1,3]. As viral transactivator proteins, Zta is unique to initiate the entire EBV lytic cascade by transactivating a series of lytic gene promoters, but Rta appears to be more effective in epithelial cells [30,31]. Increased evidences have shown that Zta initiates EBV lytic infection mainly from a methylated viral genome, whereas Rta initiates lytic infection mainly from an unmethylated genome [15,16,32,33]. Rp methylation inhibits Rta expression, however it enhances the ability of Zta to activate Rp [16]. In line with reported studies, we found that either the Rp-ZRE2 (CpG site #13-14) or/and the ZRE3 (CpG site #15) were heavily methylated in virtually all EBV-positive BL, LCL and NPC cell lines and tumors, but less methylated in Wan and B95-8 cells with basal lytic activity. A CpG methylation-free zone (three CpG sites #12-14) in Zp, located in regulatory elements YY1 and E2-2, is possibly responsible for the initial activation of *BZLF1*. Thus, Zp and Rp are regulated by both CpG methylation and cellular transcription factors, indicating the complexity of the regulation of *BZLF1 *and *BRLF1 *in EBV-associated tumors [34].

**Figure 7:**
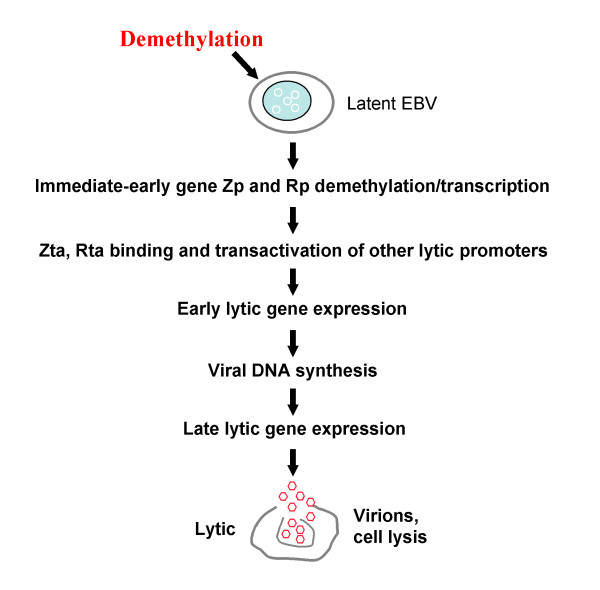
**Model of demethylation inducing EBV lytic cascade in EBV-positive tumor cells**.

DNA methylation plays a crucial role in allowing EBV to escape from the detection of host immune system. Conversely, pharmacologic reversal of viral gene methylation and activation of gene expression would resensitize host's immune surveillance or enhance its response to immunogenic viral antigens [10]. The efficacy of DNA methyltransferase inhibitors in hematologic diseases, including myelodysplastic syndromes (MDS) and acute myeloid leukemia, has been successfully evaluated by multiple clinical trials [35-37]. 5-azacytidine has been now approved as the first-line treatment of high-risk MDS [38,39]. Our group has firstly reported the achievement of successful demethylation of EBV viral antigen promoters including Cp, Wp, LMP1p, Zp and Rp, in NPC patients with azacitidine treatment [1,10-12]. When combined with other chemotherapy drugs and histone deacetylase inhibitors (HDACi), DNMT inhibitors should have even brighter perspective in the therapeutics of EBV-associated tumors (Figure 7) [40].

## Conclusions

Collectively, our study found that frequent silencing of *BZLF1 *and *BRLF1 *by hypermethylation of Zp and Rp could be reactivated by demethylation agent, resulting in the initiation of the EBV lytic cascade in EBV-associated tumors. Our study helps to understand epigenetics-related EBV pathogenesis and further develop target therapy for EBV-associated tumors.

## Competing interests

The authors declare that they have no competing interests.

## Authors' contributions

LL analyzed data and drafted the manuscript. SX and Gigi acquired data. CY and AR reviewed the manuscript. QT conceived and supervised the study, analyzed data and finalized the manuscript. All authors read and approved the final manuscript.

## Pre-publication history

The pre-publication history for this paper can be accessed here:

http://www.biomedcentral.com/1471-2407/12/125/prepub
